# Circulating miRNAs in Pediatric Pulmonary Hypertension Show Promise as Biomarkers of Vascular Function

**DOI:** 10.1155/2017/4957147

**Published:** 2017-07-27

**Authors:** Vitaly O. Kheyfets, Carmen C. Sucharov, Uyen Truong, Jamie Dunning, Kendall Hunter, Dunbar Ivy, Shelley Miyamoto, Robin Shandas

**Affiliations:** Children's Hospital Colorado, University of Colorado Anschutz Medical Campus, Aurora, CO, USA

## Abstract

**Background/Objectives:**

The objective of this study was to evaluate the utility of circulating miRNAs as biomarkers of vascular function in pediatric pulmonary hypertension.

**Method:**

Fourteen pediatric pulmonary arterial hypertension patients underwent simultaneous right heart catheterization (RHC) and blood biochemical analysis. Univariate and stepwise multivariate linear regression was used to identify and correlate measures of reactive and resistive afterload with circulating miRNA levels. Furthermore, circulating miRNA candidates that classified patients according to a 20% decrease in resistive afterload in response to oxygen (O_2_) or inhaled nitric oxide (iNO) were identified using receiver-operating curves.

**Results:**

Thirty-two circulating miRNAs correlated with the pulmonary vascular resistance index (PVRi), pulmonary arterial distensibility, and PVRi decrease in response to O_2_ and/or iNO. Multivariate models, combining the predictive capability of multiple promising miRNA candidates, revealed a good correlation with resistive (*r* = 0.97, *P*_2−tailed_ < 0.0001) and reactive (*r* = 0.86, *P*_2−tailed_ < 0.005) afterloads. Bland-Altman plots showed that 95% of the differences between multivariate models and RHC would fall within 0.13 (mmHg−min/L)m^2^ and 0.0085/mmHg for resistive and reactive afterloads, respectively. Circulating miR-663 proved to be a good classifier for vascular responsiveness to acute O_2_ and iNO challenges.

**Conclusion:**

This study suggests that circulating miRNAs may be biomarkers to phenotype vascular function in pediatric PAH.

## 1. Introduction

Pulmonary arterial hypertension (PAH) is biomechanically characterized as an increase in the resistive and reactive components of pulmonary vascular impedance [1, 2], which ultimately leads to right ventricular (RV) failure. PAH can affect adults and children, characterized by similar abnormalities in endothelial function and the intermittent formation of plexiform lesions in the vasculature, although plexiform lesions are less common in children than in adults [3, 4]. However, while survival is comparable [5] and there is no evidence that the initiating biological mechanisms are different, the underlying etiology and biomechanical progress of the disease are dissimilar between the two populations [6]. Furthermore, the extent of the effects of maturation on the pulmonary vasculature in PH is unclear. This difference in vascular biomechanics could explain why children preserve RV function longer than adults [5] and would likely results in the presence of different biomarkers in the bloodstream.

A continuing challenge in managing PAH is the availability of readily available prognostic markers that can inform clinical management. In recent decades, microRNAs (miRNA) have been recognized as important regulators of gene expression in certain cancers [7] and PAH [8, 9]. miRNAs are small noncoding RNA molecules, consisting of 12–25 nucleotides that bind to the 3'UTR of target messenger RNAs resulting in mRNA degradation or translation repression [10]. In some cases, these molecules appear in the circulation and, based on expression, offer a unique opportunity for noninvasively assessing organ function [11].

The ability to regularly and noninvasively assess disease progression could have an enormous impact on outcomes and quality of life for children suffering from PAH. The B-type natriuretic peptide (BNP) [12] and N-terminal BNP (NT-proBNP) are currently the only circulating biomarkers that are regularly utilized in the clinic. Both proteins have been shown to have prognostic [13, 14] and predictive [12, 14–16] value in adult and pediatric PH patients. However, the use of biomarkers as a complete replacement of “invasive” clinical surveillance tools has been understandably rejected [17]. On the one hand, biomarkers have been well established in symptomatic and asymptomatic heart failure [17] and showed enormous promise for predicting survival [16, 18]. On the other, correlation magnitudes between BNP and NT-proBNP and RV-PA functional characteristics are relatively low in pediatric PH [17], suggesting that additional factors are being overlooked. For example, we have shown that NT-proBNP is only correlated with pulmonary arterial impedance until the onset of interventricular septal flattening [19], thus making it an unsuitable biomarker of vascular function. Therefore, there is an urgent need for biomarkers that offer an estimate of vascular function irrespective of disease state and underlying etiology. Circulating miRNAs offer a potential solution to some of these problems as they represent varied potential biochemical markers that can sometimes be traced to specific cells in response to biomechanical stimuli, or as subordinates of other genetic and epigenetic factors [20]. The release of miRNA in response to biomechanical stimuli, when considering the aforementioned biomechanical differences between adults and children, would suggest that a different cohort of circulating miRNAs could be expected.

Circulating miRNAs can be used as functional biomarkers and provide a guide for identifying potential novel therapeutics. In fact, there is already a myriad of miRNAs that have been attributed to the development and progression of PAH [21]. However, most of these studies have been done in vitro on isolated animal cells with limited connection made to PAH functional abnormalities. Furthermore, very few studies (e.g., [21]) have investigated the role of miRNAs in the pediatric population, with none (to the best of our knowledge) focusing on pediatric PAH, whereas intergenerational differences in miRNA expression have been observed in cancer research [22]. In this study, we correlate circulating miRNAs with vascular function in the pediatric PAH population. The objective of this study is to identify circulating miRNAs that (1) correlate with functional measurements of resistive afterload, (2) correlate with functional measurements of reactive afterload, and (3) can identify patients who respond to right heart catheterization (RHC) challenge conditions with at least a 20% decrease in pulmonary vascular resistance (PVR).

## 2. Methods

### 2.1. Patient Demographic and Study Acceptance Criteria

Fourteen pediatric PAH (11 idiopathic, 1 heritable, and 2 congenital heart disease) patients (mean PA pressure > 25 mmHg) were prospectively recruited through a Colorado Multi-institutional Review Board approved study, and informed consent was obtained for all subjects. Patients underwent same-day blood analysis with a standard-of-care right heart catheterization (RHC). This study involves the retrospective analysis of this combined dataset.

### 2.2. miRNA Analysis

Two TaqMan low-density array (TLDA) cards (Thermo Fisher Sci), containing assays for 765 unique human miRNA targets (each card had unique targets), were used for each patient. Briefly, 3 *μ*L of serum was reverse transcribed using a pool of primers specific for each miRNA. To account for miRNAs expressed at very low levels, cDNA products were preamplified using specific primers. Real-time PCR reactions was performed in 384 well plates containing sequence-specific primers and TaqMan probes in the ABI7900 [21]. Here, we report the relative quantification (RQ) of targets using the comparative C_T_(ΔΔC_T_) method, where C_T_ is the cycle threshold [23]. These arrays have a high sensitivity and specificity, able to differentiate between closely related family members with a difference as minor as a single base.

### 2.3. Vascular Functional Measurements

Vascular function was assessed according to traditional clinical measures: (1) resistive component of impedance; (2) reactive component of impedance; and (3) indexed pulmonary vascular resistance (PVRi) changes under 70–100% oxygen (O_2_) and 20–40 ppm inhaled nitric oxide (iNO) challenge conditions. These parameters were acquired during the RHC protocol.

The resistive component of pulmonary vascular impedance was estimated using the equation for PVRi (1), where mPAP is the mean pulmonary arterial pressure, PCWP is the pulmonary capillary wedge pressure, and CI = CO/BSA is the cardiac index equating to the cardiac output (CO) divided by the body surface area (BSA).

(1) $$\begin{array}{c} PVRi=\frac{mPAP-PCWP}{CI}. \end{array}$$

The reactive component of pulmonary vascular impedance was indirectly estimated by PA distensibility (*D* [=] 1/mmHg) using (2) [2], which was numerically solved using Newton's method. Because simultaneous pressure and flow time-varying data were not available for these patients, which would have been the ideal dataset to compute reactive afterload, (2) was used as an indirect estimate [2].

(2) $$\begin{array}{c} mPAP=\frac{{\left[{\left(1+D\;\cdot \;PCWP\right)}^{5}+5D\;\cdot \;PVR^{\prime }\;\cdot \;CO\right]}^{1/5}-1\;}{D}. \end{array}$$

In (2), mPAP, PCWP, CO, and PVR′ are the mean pulmonary arterial pressure, pulmonary capillary wedge pressure, cardiac output, and the zero-pressure pulmonary vascular resistance. In this study, we assumed PVR = 50^∗^PVR′, estimated from the Poiseuille law: PVR/PVR′ = (*φ*_deflated_/*φ*_inflated_)^4^, where *φ* is the MPA diameter, and a typical deflated/inflated PA diameter ratio was obtained from [24] and normalized to our pediatric population.

Equation (2) was originally derived by Linehan et al. [25], who later showed that the model can be used to describe pressure and flow data in an isolated neonatal pig lung [26]. This supports the idea that this model estimates the capacitive components of the Windkessel circuit, thus making it a suitable estimate of reactive impedance.

Pulmonary vascular response to O_2_ and iNO challenges was determined based on a change in PVRi. The method consisted of measuring the relative difference in PVRi between room-air conditions and conditions after the administration of the following: (a) 70–100% O_2_; (b) inhaled nitric oxide (iNO 20–40 ppm); or (c) both. Patients with at least a 20% decrease in PVRi (*∂*PVRi) were considered “responsive.”

### 2.4. Statistical Analysis

Univariate and multivariate regression analysis was performed to identify relationships between the relative quantities (RQ) of circulating miRNAs versus patient hemodynamics. All 765 miRNA targets from the two TagMan array cards were analyzed in Matlab (Mathworks Inc., MA), with a two-tailed probability ($\begin{array}{c} P \end{array}$) less than 0.05 considered statistically significant and presented in results. All tabulated correlations were visually inspected for near-normal distribution and homoscedasticity. Bland-Altman plots were also used to compute the mean ± SD difference between the multivariate statistical model and RHC measurement.

An important note to consider is that some miRNAs were not amplified. Therefore, scores below a certain threshold for the linear phase of amplification would result in an RQ value of zero. It was not immediately clear if a patient who registered an RQ = 0 should be considered in the regression analysis, given that their score is technically categorical rather than continuous. However, they were included into the analysis because it was assumed that a low RQ, or even an RQ = 0, should be proportional to the hemodynamic variable of interest if a correlation truly exists.

Nonnormally distributed data was normalized by using Tukey's transformation [27] (*λ* = 0.3). This transformation enables even distribution of near zero values but can also be imposed on values of RQ = 0, which is not possible by logarithmic transformations.

Predictive terms for multivariate regression were found using the stepwise regression. The stepwise regression model began with 3 initial terms (miR-21, 20a, and 19b) and used entrance/exit tolerances of 0.05/0.1 on the *P* values.

The area under receiver-operating curves (ROCs) were used to identify miRNA candidates capable of classifying patient that reacted to an acute vasodilator challenge [28]. All miRNA targets were considered with area under the curve > 0.8 deemed sufficient.

## 3. Results

Fourteen pediatric PAH patients were recruited from Children's Hospital Colorado (Aurora, CO). Each patient was assigned a World Health Organization (WHO) functional classification score [29] at the time of analysis by Dr. Dunbar Ivy.  Table 1 shows a summary of patient demographics.

### 3.1. Expression of Circulating miRNAs Correlates with Resistive Impedance

**Figure 1:**
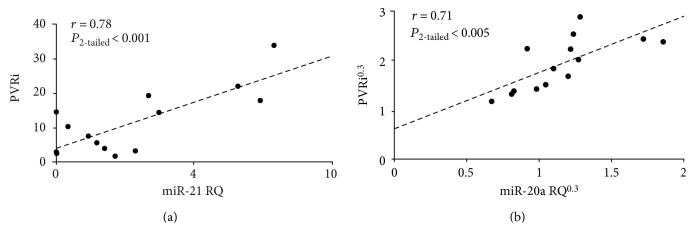
Circulating miRNAs correlated against PVRi in 14 pediatric patients. (a) miR-21 explains 61% of the variability in PVRi. (b) miR-20a explains 50% of the variability in PVRi. PVRi = indexed pulmonary vascular resistance.

Nineteen circulating miRNAs significantly (*P*_2−tailed_ < 0.05) correlated with PVRi in our cohort.  Figure 1 shows miR-21 (a) and miR-20a (b) expression was upregulated concurrently with an increase in PVRi. The correlation for these two miRNA candidates is shown because of their particularly significant association and prevalence in PAH literature (more in Discussion). The Pearson coefficient (*r*) for a complete list of miRNA candidates that correlated with PVRi is given in Table 2. Each correlation was visually inspected for homogeneity, and some distributions were normalized (indicated by superscript N) when deemed necessary.

**Figure 2:**
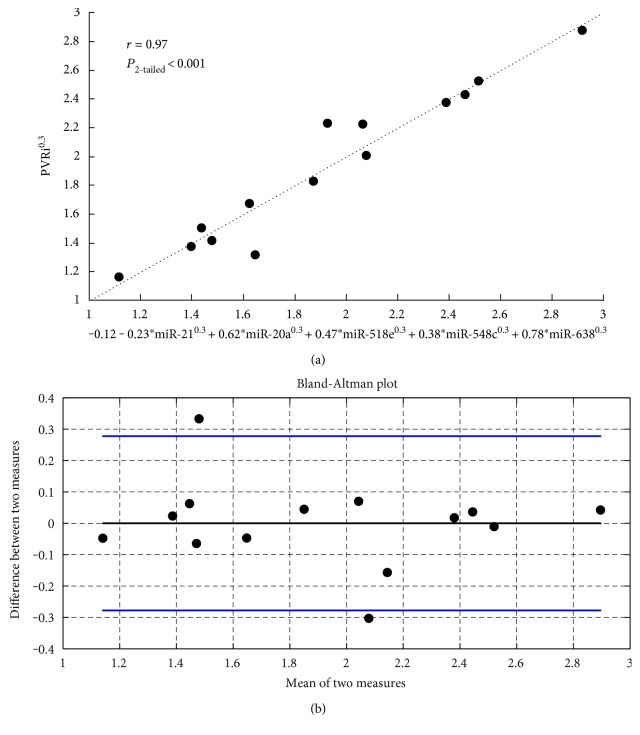
(a) Multivariate linear regression between PVRi and 5 circulating miRNAs. (b) Bland-Altman plot of the regression shown in (a).

Multivariate linear regression was used to combine the most clinically and statistically significant miRNA candidates into a single predictive model.  Figure 2 shows the resulting regression model (a) and Bland-Altman (b) plots. The multivariate model explains 94% of the variance in PVRi. The multivariate equation showed no bias, with 95% of the differences between model-predicted and RHC-measured PVRi lie within 0.27^1/0.3^ = 0.13 (mmHg−min/L)m^2^.

### 3.2. Expression of Circulating miRNAs Correlates with Reactive Impedance

**Figure 3:**
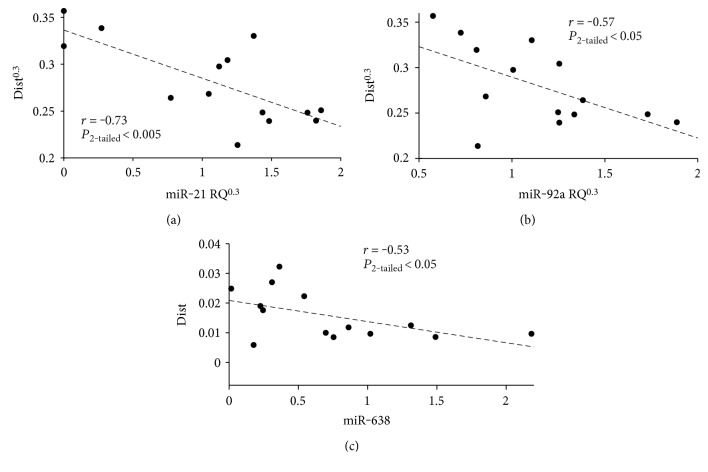
Circulating miRNAs inversely correlated with distensibility (D) in 14 pediatric patients. (a) miR-21, (b) miR-92a, and (c) miR-638 are upregulated as *D* is decreased (reactive afterload is increased).

Since there was inadequate patient data to reconstruct the entire impedance curve, we determined pulmonary artery distensibility (*D*) as a measure of reactive afterload. Out of the 765 miRNA targets considered, three candidates correlated with *D*: miR-21, miR-92a, and miR-638 (see Figure 3). Each miRNA was upregulated concurrently with an increase in reactive afterload.

**Figure 4:**
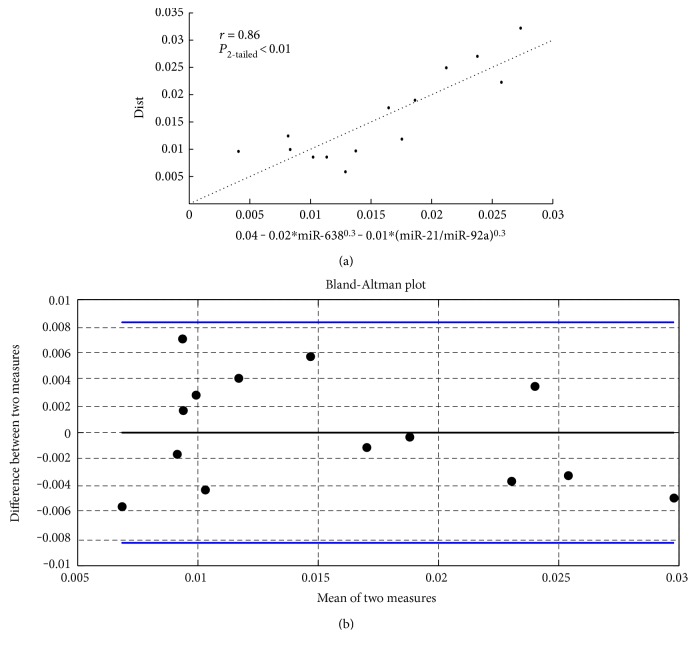
(a) Multivariate linear regression between distensibility (D) and 3 circulating miRNAs. (b) Bland-Altman plot of the regression shown in (a).

A multivariate regression combined the three aforementioned miRNAs into a single model, which notably improved the predictive capability.  Figure 4 shows the resulting regression model (a) and Bland-Altman (b) plots. The multivariate model explains 74% of the variance in *D*. The consistant bias is 0, and 95% of the differences between model-predicted and RHC-measured *D* lie within 0.0085/mmHg.

### 3.3. Circulating miRNAs Can Stratify Patients That Are Responsive to O_2_ and iNO

**Figure 5:**
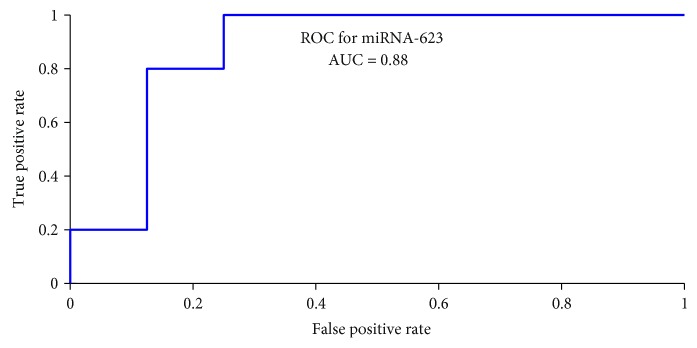
Receiver-operating curve showing miRNA-623 as the best classifier of O2 and/or iNO challenge responsiveness. Area under curve (AUC) = 0.88.

Out of 13 patients who underwent pulmonary vasodilation testing during RHC, 5/13 patients experienced a decrease in PVRi by at least 20%.  Figure 5 shows an ROC curve for miR-623, with an AUC = 0.88. While miR-623 could predict with 88% probability that a patient with a relative quantity under 1.5 (1 − specificity = 0.125; sensitivity = 0.80) will favorably respond to pulmonary vasodilator challenge, it is not capable of predicting the extent of PVRi decrease. For predicting the extent of PVRi decrease by regression, 15 circulating miRNAs were identified (see Table 3). Most notably, the downregulation in miR-627 explains 62% of the variability in the magnitude of PVRi decrease under challenge condition.

## 4. Discussion

In this study, we found a myriad of circulating miRNAs that correlate with vascular function in children with PAH. In this discussion, we characterize vascular function according to 3 criteria: (1) pulmonary vascular resistance; (2) pulmonary vascular compliance; and (3) acute vascular response to supplemental O_2_ and/or iNO. Reactive and resistive impedance account for approximately 30% [2] and 70% of the afterload, respectively. Resistive afterload, characterized as PVR, is a measure of flow energy wasted to viscous dissipation, which is not recoverable. Reactive impedance is energy transferred into distending the vasculature, considered to be recoverable as the vessel rebounds, but still increases afterload as the proximal vasculature stiffens in PAH [30]. Distensibility has been shown to predict PH severity, exercise capacity, and survival in heart failure [31, 32]. In this study, we considered distensibility to be a measure of pulmonary vascular stiffness and therefore reactive afterload.

In total, we found 32 circulating miRNAs that correlated with vascular function in 14 pediatric PAH patients. In this study, we did not focus on origin, mechanism, or target but purely explored candidates for potential clinical biomarkers. While our results show that certain miRNA candidates are sufficient predictors of invasive vascular measurements, combining several candidates into a multivariate model offers excellent predictive capabilities. In some cases, several circulating miRNAs offered more comprehensive measures of vascular function. For example, circulating miR-21, mIR-92a, and miR-638 significantly correlated with both resistive and reactive afterloads; miR-1227 and 548c were able to predict both PVRi and the extent of vascular response to vasodilators.

We focus this discussion on parallels between the circulating miRNA candidates found in our cohort and their role in PAH and cancer. We chose to include cancer literature in this discussion because it is in part mechanistically similar to PAH when considering the hyperproliferative cells involved in pulmonary vascular disease [33].

### 4.1. miRNAs Correlating with Resistive Vascular Impedance

We found statistically significant correlations between the resistive component of pulmonary vascular impedance and 19 circulating miRNAs. For brevity, we will limit our discussion to those miRNA candidates most relevant to PAH pathophysiology.

- miR-21: This has long been recognized as a key gene regulator in the development and progression of cardiovascular disease [8, 10]. When upregulated, it is associated with cardiac injury and inflammation, suppressing apoptosis and increasing proliferation in both smooth muscle cells (SMCs) and endothelial cells (ECs) [9, 34]. In human umbilical vein endothelial cells, miR-21 was upregulated during prolonged unidirectional shear stress, which decreased apoptosis and increased the secretion of vasodilators (nitric oxide). Wall shear stress, a primary factor in endothelial mechanotransduction, has been documented to decrease in PAH and is accompanied by increased PVRi [35]. Expression directionality of miR-21 is largely dependent on cell type and the etiology investigated, which sometimes reveals conflicting results. For example, monocrotaline PAH animal models and human lung tissue from idiopathic PAH patients have shown a downregulation of miR-21 [36], while hypoxia models revealed an upregulation [37]. In this study, we found upregulated miR-21 in the circulation of pediatric PAH patients occurring concurrently with an increase in resistive afterload. However, the origin of this circulating molecule remains unknown, so it could be upregulated in specific cells and downregulated in others.
- miR-19b and miR-20a: These are part of the miR-17-92 cluster, which regulate pulmonary artery SMCs and have been shown to reverse PAH [8, 9, 38]. This cluster has the potential to control multiple targets, and is upregulated in situations of reduced apoptosis and increased proliferation, which are both phenomenon shown to occur in pulmonary hypertension [8]. Specifically, miR-19b was upregulated in the hypoxic rat model [36], while miR-20a revealed an upward trend without statistical significance. Consistent with these aforementioned findings, we found circulating miR-20a to be upregulated in proportion to increasing resistive afterload in pediatric PAH.
- miR-451: This is believed to originate from the heart [39] and is upregulated in hypoxic lung tissue [36, 40–42], but one source documented a downregulation in pulmonary hypertension [43]. miR-451 was also upregulated in the monocrotaline rat pulmonary hypertension model [36]. In this study, we noticed an upregulation concurrent to an increase in PVRi, but this miRNA did not factor into the multivariate model. It is not unusual for biochemical markers that originate in the heart to be expressed in proportion to afterload. We have shown that N-terminal B-type natriuretic peptides (NT-proBNP) are correlated with resistive afterload in pediatric PH patients [19] but can be released from the myocardium of both ventricles in response to abnormal mechanical stress/strain [44, 45]. Therefore, in patients with PAH and with a flattened interventricular septum confirmed by echocardiogram, NT-proBNP levels are disproportionate with pulmonary vascular impedance. More work is needed to see if it would be possible to identify circulating miRNAs whose expression could be associated to a specific ventricle, but miR-451 could be a candidate for future studies.
- miR-223: This is believed to originate from human smooth muscle cells, and overexpression of miR-223 increases smooth muscle cell migration [46]. However, once released into the blood stream, miR-223 could have an impact on homeostasis in other organs. For example, the proteomic consequences of removing miR-223 suggest that it is a protein regulator in white blood cells [47]. Its expression is increased in heart disease [48], but no significant difference between control rats and a hypoxia-induced pulmonary hypertensive rat model was detected [36]. In this study, upregulation of miR-223 was positively correlated with resistive afterload.
- miR-146a: In this study, we found that overexpression of miR-146a in the circulation of children with PAH is correlated with an increase in PVRi. This miRNA has been shown to be an important promoter of smooth muscle cell proliferation by inhibiting Kruppel-like factor 4 expression [49]. It is induced in response to proinflammatory stimuli [50] and is highly expressed in atherosclerotic arteries, being recognized as a valuable biomarker of vascular calcification [46].
- miR-638: Expression of this miRNA has not been reported in PAH literature but is overexpressed in the circulation of children with PAH and highly correlated with both reactive and resistive afterloads in the current study. Plasma levels of miR-638 are reduced in chronic renal disease, which has a high incidence of cardiovascular complications [51]. None of the patients included in this study showed signs of renal dysfunction. miR-638 is also involved in lung destruction associated with chronic obstructive pulmonary disease, and its upregulation is highly correlated with emphysema severity [52].

### 4.2. miRNAs Correlating with a Measure of Reactive Vascular Impedance

Pulmonary distensibility, much like compliance, gradually decreases with PAH [2] due to remodeling [53]. Distensibility is a measure of the reactive component of impedance, which can contribute to as much as 30% of the afterload in the pulmonary vasculature [53].

- miR-92a: This is a component of the miR-17–92 cluster [48], which was discussed above. It has been linked to PAH but did not show a significant difference between patients and control subjects [54]. Noteworthy, in the hypoxic mouse and monocrotaline rat pulmonary hypertensive models, miR-92a antagomirs reduced PA muscularization [10], which could partially explain its correlation to the reactive component of impedance in this study. However, directionality between the two rat pulmonary hypertensive models were conflicting. In our cohort, upregulation of miR-92a is associated with an increase in reactive afterload and general disease progression.
- Circulating miR-21 and miR-638 were discussed above.

### 4.3. Circulating miRNAs Correlated with Decreased PVRi in Response to O_2_ and/or iNO

Sixteen circulating miRNAs were identified as potential biomarkers of a response to vasodilatory challenge. Once candidate could stratify patients according to responsive versus nonresponse, while 15 had no stratification capability but correlated with the extent of PVRi decrease. We show preliminary evidence to suggest an 88% probability that a responsive patient will have an RQ miR-623 over 1.5 compared with a nonresponsive patient [28]. Thus, this makes miR-623 a “good” classifier of patients who will experience at least a 20% reduction in PVRi when administered with iNO or supplemental O_2_. To our knowledge, this is the first study attributing clinical utility to miR-623 in PAH. In other possibly connected research, miR-623 was upregulated in connection to T cell intracellular antigen protein depletion [55] in HeLa cells, where T cells have been shown to produce NO via endothelial NO synthase within minutes of binding to antigen [56]. Nevertheless, the RQ of miR-623 did not correlate with the extent of PVRi change (∂PVRi). Of the 15 candidates that correlated with the extent of PVRi decrease, miR-627 predicted 62% of the variability in ∂PVRi but has not been implicated in PAH. In fact, most of the listed miRNAs do not readily appear in PAH literature. Although, miR-212 has been shown to be upregulated in failing hypertrophic hearts [57].

### 4.4. Clinical Translation

The miRNAs presented in this manuscript, identified as significant measures of pulmonary arterial impedance and reactivity to supplemental O_2_ and iNO, represent potential candidates as biomarkers that could comprehensively phenotype vascular functional preservation in PAH disease. Both single circulating miRNAs and multivariate combinations appear to be potential candidates for noninvasively evaluating vascular function by blood biochemical testing in pediatric PAH. If realized, this could lead to significant improvements in medical management and outcomes.

### 4.5. Basic Science Translation

In order to use these findings to identify novel therapeutic targets, the cellular targets of the listed miRNAs must be identified. This study was intended to identify circulating miRNAs with potential as a biomarker of PAH disease and outcome, though we were unable to determine the origin, target organs, or specific mechanisms of these miRNAs. This comprehensive assessment of circulating miRNAs as a biomarker in pediatric PAH provides an important framework for future studies to address the many knowledge gaps in pediatric PAH research.

### 4.6. Limitations and Future Work

This study had some important limitations. (1) The small sample size could be particularly erroneous for receiver-operating curve classification techniques. This also prevented us from assessing acute vascular reactivity according to the Barst or Sitbone criteria [58, 59]. (2) Array results are without real-time- (RT-) PCR validation. This is a common limitation in such exploratory studies, but future work will validate these findings with RT-PCR and use the miRNAs identified in this study as a guide. (3) In depth discussion was primarily limited to those miRNAs with previous relevance to PAH patients, animal models, and isolated cell studies. The other miRNAs that demonstrated significant associations with vascular function as outlined in Tables 2 and 3 likely have equal importance with respect to biomarker potential in this population even though they do not readily appear in PAH literature.

Future work will build on this exploratory dataset to expand our cohort and link circulating miRNAs with ventricular function and outcomes. Furthermore, we will investigate the impact of common PH therapy, gender, and development on changes in circulating miRNA expression. In fact, it is likely that the development from childhood to young adulthood could alter the cohort of biomarkers in the circulation, which requires additional studies.

## 5. Conclusion

Serum miRNA arrays of 14 pediatric patients with PAH identified 32 circulating miRNA candidates that correlate with vascular function (e.g., impedance and response to acute O_2_ and/or iNO challenge). Considering the lifetime accumulated risk and cost associated with routine right heart catheterizations and other advanced imaging modalities currently used to monitor pediatric PAH, miRNAs show promise as possible noninvasive and low-risk biomarkers. Although more research is required and the sample size must be increased, this could offer clinicians a potential tool for routinely evaluating disease progression and therapeutic efficacy, and possible future therapeutic targets. Some of the identified biomarkers have been well documented in PAH literature, some are implicated in overproliferative mechanisms associated with cancer, while others are being connected to PAH for the first time. As previously mentioned, a larger sample size is needed to establish these circulating miRNAs as biomarker candidates, and origin/target must be identified to consider miRNA-based therapy. However, their identification in a pediatric PAH population provides a framework for future work.

## Figures and Tables

**Table 1:** Summary of patient demographics.

Patient	BSA	PVRi	Weight	WHO	Age (mo.)	Gender	Etiology
1	0.95	2.48	24.6	I	108	M	Idiopathic
2	1.83	19.2	63.9	III	213	M	Idiopathic
3	1.74	2.89	63.5	I	278	F	Idiopathic
4	0.82	17.78	19.9	II	60	F	Idiopathic
5	0.99	14.49	27.6	I	96	F	Heritable
6	1.42	1.66	48.2	I	144	M	Idiopathic
7	1.6	14.32	59.0	III	317	F	Idiopathic
8	1.72	21.94	66.7	III	329	F	Idiopathic
9	0.75	3.89	19.5	I	71	M	Idiopathic
10	1.51	33.78	49.5	III	240	F	CHD
11	1.44	5.54	44.2	II	204	M	Idiopathic
12	1.6	7.47	57.1	III	192	M	CHD
13	0.98	3.18	25.6	I	83	F	Idiopathic
14	0.99	10.21	26.2	II	108	F	Idiopathic

CHD: congenital heart disease.

**Table 2:** 19 miRNAs identified as correlating against PVRi in a pediatric PAH population. Before performing correlation, the distribution of the miRNA in question was normalized (subscripted by N) by performing Tuckey's transformation (discussed in Methods) and each correlation was visually inspected for homogeneity of variance. PVRi = indexed pulmonary vascular resistance.

miRNA	Pearson's coefficient (*r*)	*P*_2-tailed_
miR-21	0.78	<0.001
miR-19b	0.62	0.018^N^
miR-146a	0.62	0.018^N^
miR-20a	0.71	<0.005^N^
miR-223	0.58	0.030^N^
miR-375	−0.53	0.051^N^
miR-451	0.56	0.037^N^
miR-484	0.55	0.042^N^
miR-486	0.57	0.033
miR-548c	0.63	0.016^N^
miR-638	0.68	0.007^N^
miR-657	0.65	0.012^N^
miR-661	0.70	0.005^N^
miR-1227	0.59	0.026^N^
miR-16	0.53	0.051^N^
miR-489	−0.55	0.042^N^
miR-518e	0.69	0.006^N^
miR-92a	0.55	0.042^N^
miR-615	0.54	0.046^N^

**Table 3:** 15 miRNAs identified as correlating against the maximum change in PVRi between baseline and challenge conditions in a pediatric PAH population. Before performing correlation, the distribution of some miRNAs in question was normalized by performing a logarithmic transformation and each correlation was visually inspected for homogeneity of variance.

miRNA	Pearson's coefficient (*r*)	*P*_2-tailed_
miR-299-5p	−0.72	0.0037
miR-323-3p	−0.70	0.0053
miR-627	−0.79	0.0008
miR-636	−0.65	0.0119
miR-520h	0.58	0.0297
miR-559	0.56	0.0373
miR-299-3p	−0.64	0.0137
miR-323-3p	−0.73	0.0030
miR-212	−0.56	0.0373
miR-548c	−0.68	0.0075
miR-486-3p	−0.65	0.0119
miR-496	−0.71	0.0044
miR-618	−0.58	0.0297
miR-432	−0.61	0.0205
miR-1227	0.59	0.0264
